# Description of four new terrestrial diatom species from *Luticola* and *Microcostatus* genera from South Africa

**DOI:** 10.3897/phytokeys.181.65326

**Published:** 2021-09-14

**Authors:** Mateusz Rybak, Natalia Kochman-Kędziora, Łukasz Peszek

**Affiliations:** 1 University of Rzeszów, Department of Agroecology and Forest Utilization, Ćwiklińskiej 1a, 35-601, Rzeszów, Poland University of Rzeszów Rzeszów Poland; 2 University of Rzeszów, Department of Ecology and Environmental Protection, Zelwerowicza 4, 35–601, Rzeszów, Poland University of Rzeszów Rzeszów Poland

**Keywords:** Bacillariophyceae, bryophytes, *
Luticola
*, *
Microcostatus
*, new species, South Africa

## Abstract

The knowledge about terrestrial diatom assemblages in southern Africa is rather limited, despite a long history of diatom research in this area. Terrestrial habitats are places of characteristic diatom floras, dominated by species resistant to desiccation which are able to thrive in conditions of limited water availability. The presented work expands the knowledge about these unique habitats. During the study on terrestrial moss-inhabiting diatoms from Western Cape Province (South Africa), four taxa with a unique set of valve features have been found and described herein as new species, based on light and scanning electron microscopy. These new species are: *Luticolamicrocephala* M. Rybak, Peszek & Kochman-Kędziora, **sp. nov.**, *Luticolaasymmetrica* M. Rybak, Kochman-Kędziora & Peszek, **sp. nov.**, *Luticolaterrestris* Kochman-Kędziora, M. Rybak & Peszek, **sp. nov.** and *Microcostatusmeridionalis* Peszek, M. Rybak & Kochman-Kędziora, **sp. nov.** Algal community composition had low species richness (9–15 taxa per sample) and samples were dominated by: *Humidophilacontenta*, *Nitzschiabrevissima* and Eunotiaaff.pseudominor. The three new *Luticola* species formed numerous populations, exceeding 10% of the share, whereas *Microcostatusmeridionalis* reached 5.4%. Basic morphological data of associated diatom flora together with detailed micrographs are also provided.

## Introduction

Research on African diatoms has a long tradition, with the first works dating back to the 19th century (i.e. Ehrenberg 1845; Cleve 1881). The dynamic development of diatomological research took place mainly in the second half of the 20th century. Research from diverse habitats was carried out by four scientists: B.J. Cholnoky, M.H. Giffen, F.R Schoeman and R.E.M. Archibald, whose efforts are the foundation of more recent studies (i.e. Cocquyt et al. 2017; Cocquyt and Taylor 2019; Ridder and Taylor 2020). The general diatom diversity of inland waters from southern Africa was studied by Cholnoky (e.g. Cholnoky 1960, 1962, 1966), who also described many new species (Cholnoky 1954, 1955, 1956, 1957, 1966). On the other hand, Giffen, in his works from the mid-1960s, dealt mainly with marine and estuarine diatoms (i.e. Giffen 1963, 1967, 1970, 1973, 1976), as well as some freshwater species from the Eastern Cape Region (Giffen 1966). Undoubtedly, the greatest contribution to understanding both the taxonomy and ecology of diatoms was made by Schoeman and Archibald, who, for over 15 years, published together over 20 works on diatom flora in southern Africa, supplemented by hand-drawings (i.e. Schoeman 1969, 1970; Archibald 1966) and, in subsequent years, also with microphotographs of the observed species (i.e. Schoeman and Archibald 1986a, 1986b, 1986c, 1986d; Archibald and Schoeman 1984, 1985). More recent researchers continued to work on taxonomy (i.e. Cocquyt 2006; Cocquyt et al. 2013, 2014, 2016; Taylor et al. 2014a, b), but also concentrated on ecological monitoring (i.e. Bellinger et al. 2006; Taylor et al. 2007b, c). There are two elaborate reports of the most common diatom species from the Congo and Zambezi Basins (Taylor and Cocquyt 2016) and South Africa (Taylor et al. 2007a). In recent years, many new species were described from different parts of Africa. Various studies confirm the presence of a unique diatom diversity, composed of rare and endemic diatoms species (Cocquyt et al. 2013, 2014, 2017; Cocquyt and Ryken 2017; Taylor et al. 2014b).

Despite a long history of diatom research in southern Africa, the knowledge about terrestrial diatom assemblages is rather limited (Taylor et al. 2010, 2014a; Van de Vijver et al. 2010). Although most of the studies focused on marine, freshwater and brackish diatoms, there are still some unexplored aerial habitats. A study of terrestrial moss-inhabiting diatom communities, conducted by Van de Vijver et al. (2010), resulted in the description of one species, *Muelleriataylorii* Van de Vijver & Cocquyt from Drakensburg, Freestate Province, South Africa.

Two genera are mainly considered to occur in terrestrial habitats, like mosses, rocks or soils, the genus *Luticola* and genus *Microcostatus* (Round et al. 1990; Johansen and Sray 1998). The genus *Luticola* D.G. Mann (Round et al. 1990) was distinguished from *Navicula* to accommodate species included in Naviculaesect.Punctatae with Luticolamutica (Kütz.) D.G. Mann as generitype. So far, over 200 species have been documented worldwide (Guiry and Guiry 2021). A monograph, summarising the entire genus (Levkov et al. 2013), presented a detailed and extensive revision of the *Luticola* genus, including more than 20 species observed from Africa. Still, more recent studies show that the diversity of the genus *Luticola* is underestimated and many new species have been described from Europe (Levkov et al. 2017), Asia (Liu et al. 2017; Glushchenko et al. 2017; Lokhande et al. 2020), South America (Bąk et al. 2017; Straube et al. 2017; Simonato et al. 2020; Peszek et al. 2021), Madagascar (Bąk et al. 2019) and Antarctic Region (Zidarova et al. 2014; Kohler et al. 2015; Chattová et al. 2017; Kochman-Kędziora et al. 2020). The genus *Luticola* is highly diverse in valve morphology. Species of this genus are also widespread in brackish, freshwater and terrestrial ecosystems. However, the genus is ecologically characterised as being aerophilous, often noted from sites in splash zones, soil and amongst mosses (Round et al. 1990; Kociolek et al. 2017). It also shows a high degree of endemism (Kociolek et al. 2017; Lokhande et al. 2020).

The genus *Microcostatus*, described by Johansen and Sray (1998), encompasses small naviculoid species with two longitudinal depressions next to the central sternum, a simple raphe system and external microcostae. Until now, this small genus has only 24 species documented worldwide (Guiry and Guiry 2021). Taylor et al. (2010) listed 23 taxa, including three described from South Africa. More recently, papers described additional six species: *Microcostatusedaphicus* Stanek-Tarkowska, Noga, Wetzel & Ector and *M.aerophilus* Stanek-Tarkowska, Noga, Wetzel & Ector from Europe (Stanek-Tarkowska et al. 2016), *M.salinus* Li & Suzuki (Li et al. 2016) and *M.werumii* Metzeltin, Lange-Bertalot & Soninkhishig (Metzeltin et al. 2009) from Asia and two other species: *M.australoshetlandicus* Van de Vijver, Kopalová, Zidarova & E.J. Cox and *M.elisabethianus* Van de Vijver & Ector from Antarctic and Sub-Antarctic Region (Van de Vijver et al. 2013; Van de Vijver and Ector 2019).

The present paper aims to contribute information on the distribution and environmental preferences of terrestrial diatoms in South Africa. This is the first paper providing information about moss-inhabiting diatom assemblages from Western Cape Province, South Africa. Three taxa from the genus *Luticola* and one from *Microcostatus*, which cannot be identified using currently available literature, were observed during the study. Additional analysis, based on detailed light and scanning electron microscopy, showed a set of unique features allowing us to describe them as new species. Additional comparison with the most similar taxa is also provided.

## Methods

The study area is situated in the Western Cape Province, on the south-western coast of South Africa. The climatological conditions in the Western Cape Province are influenced by both the Indian and Atlantic oceans. Most of the Province is considered to have a Mediterranean climate characterised by cool and wet winters (June–August), whereas summers (December–February) are warm and dry (Ndlovu et al. 2019). The Western Cape is widely-known for its high level of endemism in terrestrial plants, especially fynbos vegetation and naturally-occurring acidic water (Brown and Magoba 2009).

For this study, the moss samples were collected in September 2018 from three different study sites in Western Cape Province, South Africa. Two sites were located on the edge of the forest by the Prince Alfred’s Pass. The third site was situated in the Jonkershoek Nature Reserve, where the sample was collected from moss growing on the rock (Table 1). The samples were collected under permit No: CN35-285316 issued by CapeNature, OP 3570/2018.

**Table 1.** Sampling site characteristics.

Sample number	Locality	Coordinates	Altitude (m a.s.l.)	Substrate	Sampling date
2018/424	Prince Alfreds’s Pass, Knysna, Western Cape, South Africa	33°58.458'S, 23°08.811'E	428	terrestrial moss collected from soil	20 September 2018
2018/425	Prince Alfreds’s Pass, Knysna, Western Cape, South Africa	33°58.475'S, 23°08.797'E	428	terrestrial moss collected from rock	20 September 2018
2018/426	Jonkershoek Nature Reserve, Western Cape, South Africa	33°59.695'S, 18°58.726'E	397	terrestrial moss collected from rock	02 September 2018

In the laboratory, the moss samples were digested in sulphochromic mixture – a mixture of concentrated sulphuric acid and potassium dichromate in order to obtain clean diatom frustules. To remove the sulphochromic mixture, the material was centrifuged at 2500 rpm with distilled water. The cleaned diatom suspension was dried on microscope cover-slips and mounted in the synthetic Naphrax Brunel Microscopes Ltd., Chippenham, UK (refractive index 1.73). Diatoms were identified and counted under a light microscope (LM) Carl Zeiss Axio Imager A2, equipped with a 100× Plan Apochromatic objective with differential interference contrast (DIC) for oil immersion. Diatom images were captured with a Zeiss AxioCam ICc5 camera. For the observations in a scanning electron microscope (SEM), the samples were applied to a polycarbonate membrane filter with a 3 µm mesh, attached to aluminium stubs and sputter-coated with 20 nm of gold, using a turbo-pumped Quorum Q 150T ES coater. Diatoms were analysed using a Hitachi SU8010 SEM. The storage locations of holotype and isotype slides (Diatom Collections) for each of the newly-described species are indicated in the descriptions. Diatom terminology and identification was based on the following references: Round et al. (1990); Taylor et al. (2007a, 2010); Metzeltin and Lange-Bertalot (2002); Levkov et al. (2013); Taylor and Cocquyt (2016); Houk et al. (2017); Lange-Bertalot et al. (2017). Species composition and relative abundance of taxa in diatom assemblages were determined by counting 300 valves in each sample.

## Results

### Descriptions of new species

#### Division: Bacillariophyta Haeckel

**Class: Bacillariophyceae Haeckel**

**Subclass: Bacillariophycidae D.G. Mann**

**Order: Naviculales Bessey**

**Family: Diadesmidaceae D.G. Mann**

#### Genus: *Luticola* D.G. Mann in Round et al., 1990

##### Luticola microcephala

Taxon classificationAnimaliaNaviculalesDiadesmidaceae

M. Rybak, Peszek & Kochman-Kędziora sp. nov.

A8F73988-55D2-508C-9660-DAE51E726B65

Fig. 1

###### Holotype.

Slide no. 20-093 stored at the South African National Diatom Collection (SANDC) at North-West University, Potchefstroom, South Africa.

###### Isotype 1.

Slide no. 27525 and unmounted material with the same number at the Szczecin Diatom Collection (SZCZ) hosted by the University of Szczecin.

###### Isotype 2.

Slide no. 2018/426 and unmounted material with the same number at the University of Rzeszów, Poland.

###### Type locality.

Jonkershoek Nature Reserve, Western Cape, South Africa, 33°59.695'S, 18°58.726'E, *leg.* W. Morek and B. Surmacz, *20.09.2018*.

###### Etymology.

The specific epithet refers to the size and shape of valve apices.

**Figure 1.**
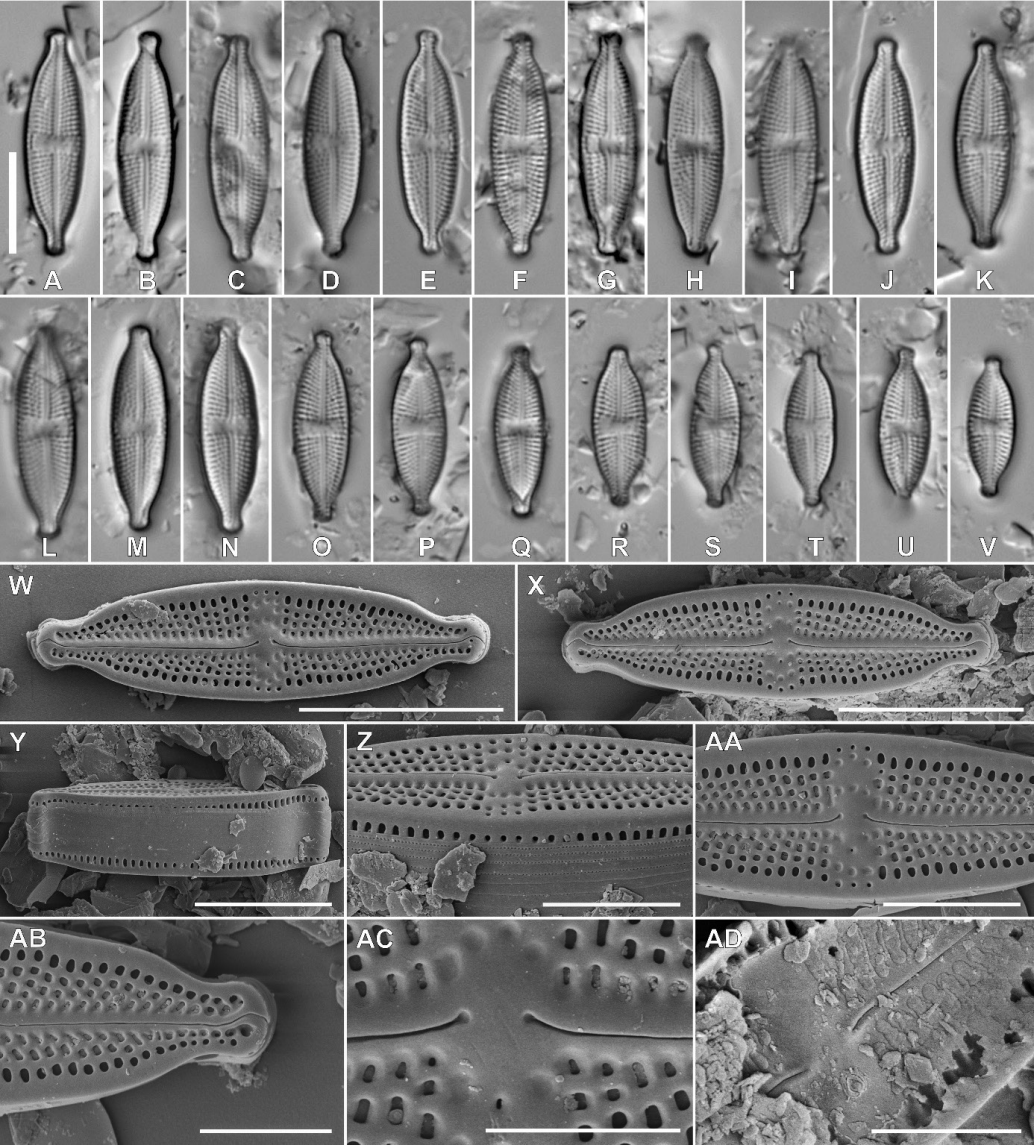
**A–AD** Holotype population of *Luticolamicrocephala* M. Rybak, Peszek & Kochman-Kędziora, sp. nov. **A–V**LM images of valve views **W–AD**SEM images **W–X** external view of valves **Y** external view of frustule girdle view **Z** partial valve view and girdle view of middle frustule section **AA** central area with several shallow depressions **AB** raphe structure with detailed view on distal raphe endings **AC** proximal raphe endings **AD** internal view of valve. Scale bars: 10 µm **(A–Y)**, 5 µm **(Z–AA)**, 4 µm **(AB)**, 3 µm **(AC, AD)**.

**Description. LM** (Fig. 1A–V). Valves linear-lanceolate to lanceolate with convex margins and clearly protracted, capitate, small apices, rectangular in girdle view. The width of apices is approximately one third of the valve width. Valve dimensions (n = 25): length 14.0–24.0 μm, width 4.5–6.6 μm. Axial area linear, narrow. An isolated pore present in the central area, located halfway between valve margin and proximal raphe endings. Central area rectangular to slightly bow-tie-shaped and asymmetric, bordered on both sides with 3–4 areolae. Irregularly-scattered areolae and shallow depressions present in the central area. Raphe branches straight, proximal raphe endings deflecting away from isolated pore. Transapical striae radiate throughout, 19–22 in 10 μm.

**Description. SEM** (Fig. 1W–AD). Externally, striae composed of 1–4 areolae, decreasing from 3–4 in striae next to the central area to only one next to the apices. Areolae elongated, becoming larger towards the valve margin (Fig. 1W, X, AA, AB). On both sides, the central area bordered by 3 round, isolated areolae. Several ghost areolae present in the central area (Fig. 1AA, AC). Raphe branches positioned on the slightly raised sternum (Fig. 1Z). Proximal raphe endings shortly bent away from the small, round isolated pore (Fig. 1 AA, AC). Distal raphe fissures hooked, first deflected towards the same side as the proximal raphe endings, then hooked towards the opposite side, continuing on to the mantle (Fig. 1W, X, AB). Single row of large, usually elongated areolae present on the mantle (Fig. 1Y). Only close to the apices and in the central part of the valve, areolae becoming smaller and rounded (Fig. 1Y, Z). Copulae numerous with 1 to 3 rows of areolae (Fig. 1Z). Internally, areolae occluded by hymenes forming continuous strip (Fig. 1AD). Isolated pore opening rounded, covered by a lipped slit (Fig. 1AD). Longitudinal channel visible internally along valve edges.

##### Luticola asymmetrica

Taxon classificationAnimaliaNaviculalesDiadesmidaceae

M. Rybak, Kochman-Kędziora & Peszek sp. nov.

91551BEF-3C84-52FD-9559-8D310116F316

Fig. 2

###### Holotype.

Slide no. 20-091 stored at the South African National Diatom Collection (SANDC) at North-West University, Potchefstroom, South Africa.

###### Isotype 1.

Slide no. 27523 and unmounted material with same number stored at the Szczecin Diatom Collection (SZCZ) hosted by the University of Szczecin.

###### Isotype 2.

Slide no. 2018/425 and unmounted material with the same number at the University of Rzeszów, Poland.

###### Type locality.

Prince Alfreds’s Pass, Knysna, Western Cape, South Africa, 33°58.475'S, 23°08.797'E, *leg.* W. Morek and B. Surmacz, *20.09.2018*.

###### Etymology.

The specific epithet refers to the species asymmetry in valve outline and proximal raphe endings.

**Figure 2.**
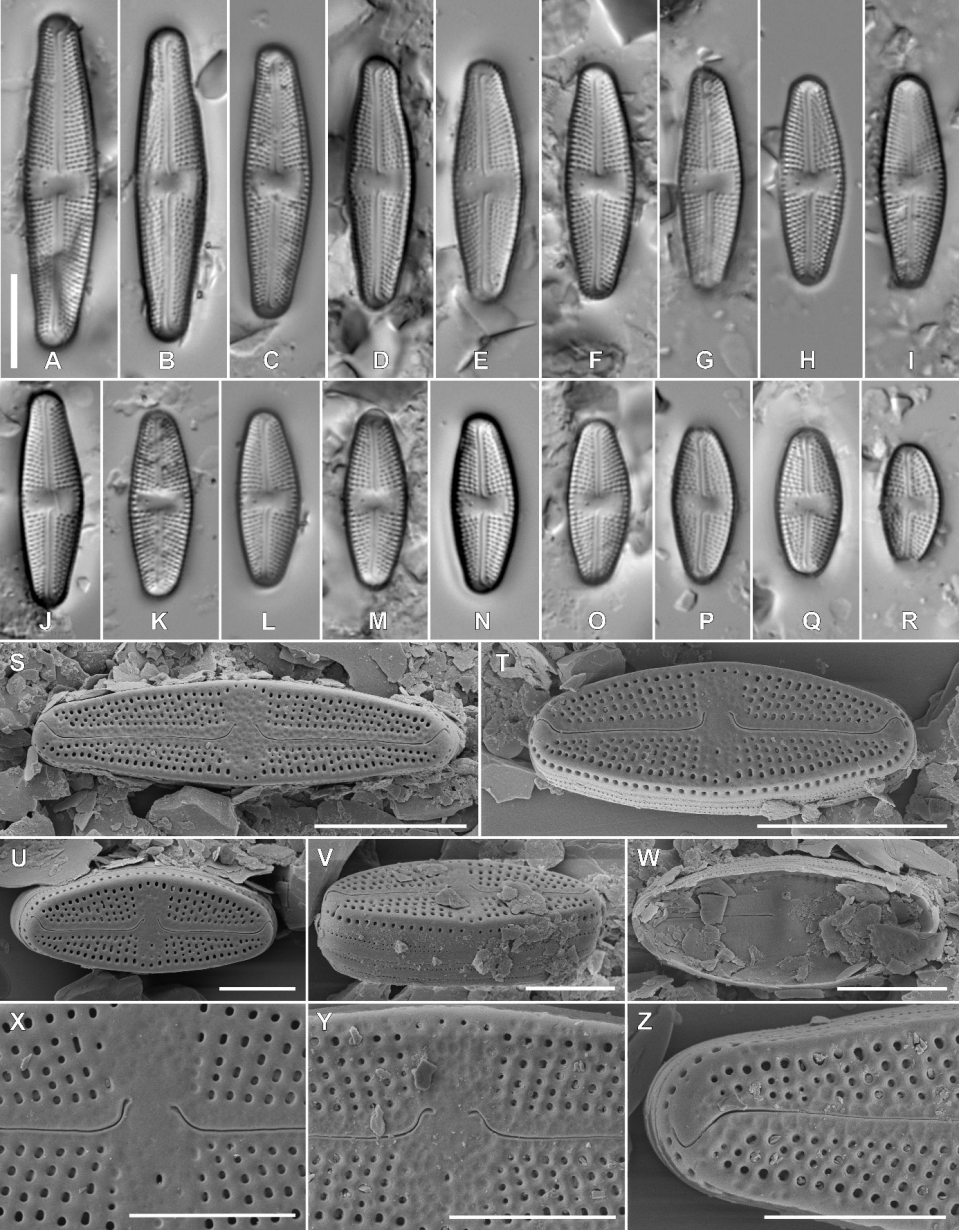
Holotype population of *Luticolaasymmetrica* M. Rybak, Kochman-Kędziora & Peszek, sp. nov. **A–R**LM images of valve views **S–Z**SEM images **S–V** external view of an entire valve **W** internal valve face view of an entire valve **X–Y** different shape of proximal raphe endings **Z** distal raphe endings. Scale bars: 10 µm (**A–T**), 5 µm (**U–Z**).

**Description. LM** (Fig. 2A–R). Valves weakly asymmetric with convex margins, rectangular in girdle view. Larger valves lanceolate with protracted, subrostrate apices; smaller valves elliptic–lanceolate to rhombic lanceolate with broadly-rounded apices. Valve dimensions (n = 25): length 12.5–36.0 μm, width 6.0–8.0 μm. Axial area linear, slightly widening towards both the central area and the apices. Central area bow-tie-shaped, bordered by 3–5 shortened striae. One isolated pore present in the central area. Raphe branches straight. Proximal raphe endings unilaterally deflected away from the isolated pore. Terminal raphe fissures elongated, hooked. Striae radiate throughout, 17–20 in 10 µm.

**Description. SEM** (Fig. 2S–Z). Striae composed of 2–5 elongated areolae. Areolae close to the valve margin larger (Fig. 2S, T, Z). Usually 3–5 isolated areolae positioned on both sites in central area, close to the valve margin. Small, round isolated pore located in the central area, halfway between the valve margin and proximal raphe endings (Fig. 2S–U, X, Y). In some specimens, small, irregular depressions present on the valve face producing uneven appearance of the valve face (Fig. 2S, X–Z). Raphe branches straight. Proximal raphe fissures long, unilaterally deflected to the side opposite to stigma and expanded into small pores. In some specimens, proximal raphe endings asymmetrical (Fig. 2T, U, X, Y). Distal raphe fissures hooked towards opposite side, terminating shortly before valve edge (Fig. 2S–V, Z). Distal raphe fissures interrupting row of areolae on the valve mantle (Fig. 2Z). One row of round areolae present on the valve mantle (Fig. 2T–V). Copulae with 2 rows of areolae (Fig. 2V). Internally, areolae occluded by hymenes forming irregular strip. Isolated pore opening rounded, covered by a lipped slit (Fig. 2W).

##### Luticola terrestris

Taxon classificationAnimaliaNaviculalesDiadesmidaceae

Kochman-Kędziora, M. Rybak & Peszek sp. nov.

51AA4AE4-5DEE-5A58-B7D5-E9EAEB519091

Fig. 3

###### Holotype.

Slide no. 20-092 stored at the South African National Diatom Collection (SANDC) at North-West University, Potchefstroom South Africa.

###### Isotype 1.

Slide no. 27524 and unmounted material with same number at the Szczecin Diatom Collection (SZCZ) hosted by the University of Szczecin.

###### Isotype 2.

Slide no. 2018/424 and unmounted material with the same number at the University of Rzeszów, Poland.

###### Type locality.

Prince Alfreds’s Pass, Knysna, Western Cape, South Africa, 33°58.458'S, 23°08.811'E, *leg.* W. Morek and B. Surmacz, *20.09.2018*.

###### Etymology.

The specific epithet refers to the terrestrial habitat from where the new species is described.

**Figure 3.**
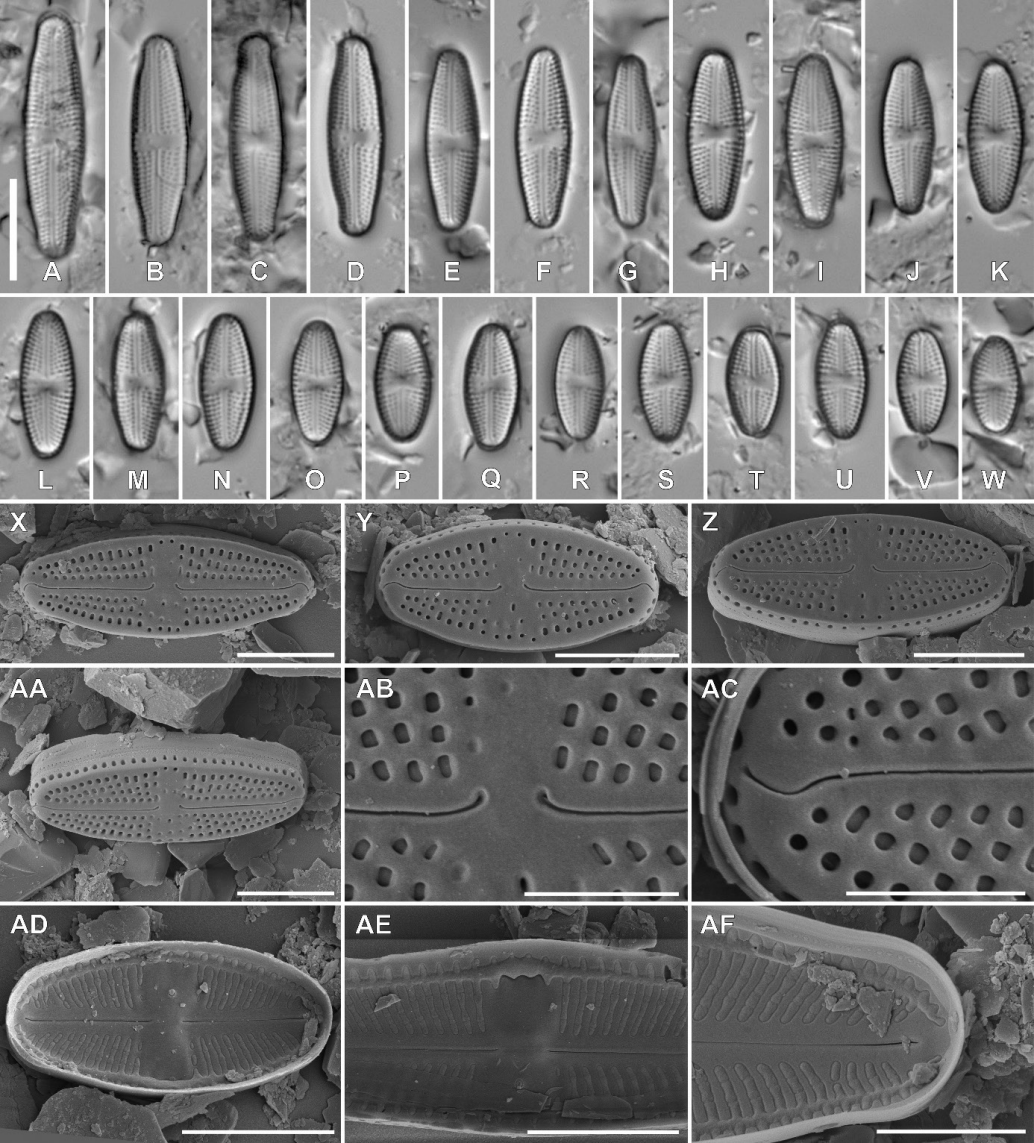
Holotype population of *Luticolaterrestris* Kochman-Kędziora, M. Rybak & Peszek, sp. nov. **A–W**LM images of valve views **X–AF**SEM images **X–AA** external view of an entire valve with several ghost areolae in the central area **AB** proximal raphe endings **AC** distal raphe endings **AD** internal view of an entire valve **AE** central area, internal view **AF** valve apex, internal view. Scale bars: 10 µm (**A–W**), 5 µm (**X–AA, AD, AE**), 4 µm (**AF**), 2 µm (**AB, AC**).

**Description. LM** (Fig. 3A–W). Larger valves lanceolate with weakly-protracted apices; smaller valves rhombic-lanceolate, rectangular in girdle view. Apices usually rounded, in larger valves, slightly subcapitate. Valve dimensions (n = 25): length 8.0–28.5 μm, width 4.4–6.1 μm. Axial area linear, slightly widening towards both the central area and the apices. Central area bow-tie-shaped, often asymmetrical, bordered by shortened striae. One isolated pore present in the central area. Raphe branches straight. Proximal raphe endings unilaterally deflected away from the isolated pore; terminal raphe fissures elongated and hooked. Striae radiate throughout, 20–23 in 10 μm.

**Description. SEM** (Fig. 3X–AF). Striae composed of 2–4 transapically elongated areolae becoming larger towards the valve margins (Fig. 3X–AA). One elongated stigma present, positioned in between the proximal raphe endings and the valve face margin (Fig. 3X–Z). Ghost areolae often present, mainly on a stigma-bearing site (Fig. 3X, AA). Raphe branches straight. Externally, proximal raphe endings deflected away from the isolated pore-bearing side with small drop-like endings (Fig. 3AB). Terminal raphe fissures clearly elongated, first curved to the same side as the proximal raphe fissures, then slightly bent to the isolated pore-bearing side. Terminal raphe fissures are towards the valve apices, terminating on the valve face/mantle junction, well after the final row of areolae (Fig. 3AC). Valve mantle bearing a single row of rounded areolae (Fig. 3Z, AA). Copulae with single row of areolae (Fig. 3AA). Internally, areolae occluded by hymenes, forming a continuous strip across each stria (Fig. 3AD–AF). Internal isolated pore opening rounded, covered by a lipped slit. Proximal and terminal raphe endings weakly deflected towards the pore (Fig. 3AE), the latter terminating on to small helictoglossae (Fig. 3AF). Longitudinal channel visible along valve edges (Fig. 3AE, AF).

#### Division: Bacillariophyta Haeckel

**Class: Bacillariophyceae Haeckel**

**Subclass: Bacillariophycidae D.G. Mann**

**Order: Naviculales Bessey**

**Family: Naviculaceae Kützing**

**Genus: *Microcostatus* Johansen & Sray, 1998**

##### Microcostatus meridionalis

Taxon classificationAnimaliaNaviculalesSellaphoraceae

Peszek, M. Rybak & Kochman-Kędziora sp. nov.

910D55F3-F3F3-5BD6-A976-8C0CD2FC9221

Fig. 4

###### Holotype.

Slide no. 20-093 stored at the South African National Diatom Collection (SANDC) at North-West University, Potchefstroom, South Africa.

###### Isotype 1.

Slide no. 27525 and unmounted material with same number at the Szczecin Diatom Collection (SZCZ) hosted by the University of Szczecin.

###### Isotype 2.

Slide no. 2018/426 and unmounted material with the same number at the University of Rzeszow, Poland.

###### Type locality.

Jonkershoek Nature Reserve, Western Cape, South Africa, 33°59.695'S, 18°58.726'E, *leg.* W. Morek and B. Surmacz, *20.09.2018*.

###### Etymology.

The name refers to the area from where the new species is described (lat. *meridional* – southern).

**Figure 4.**
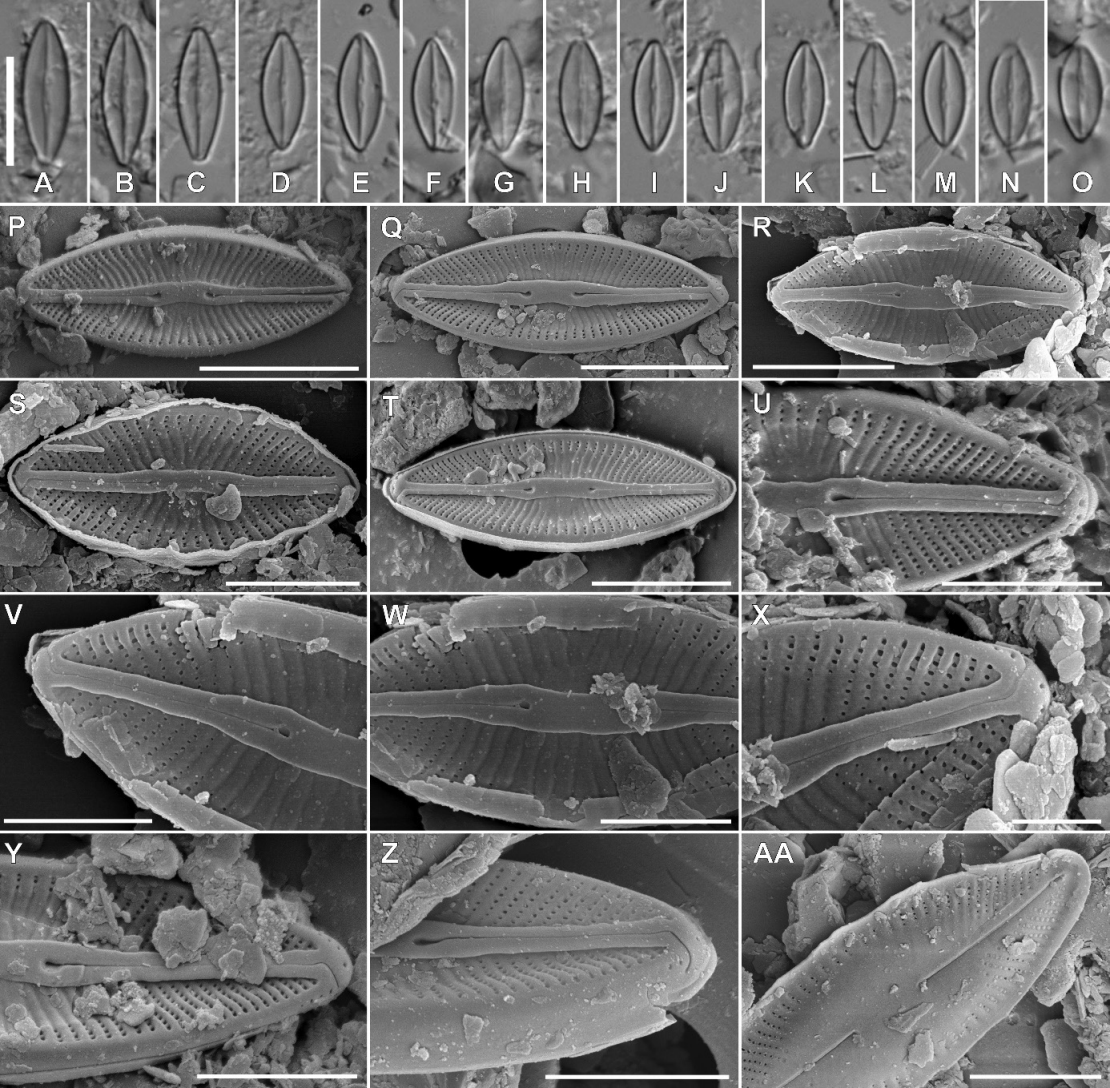
Holotype population of *Microcostatusmeridionalis* Peszek, M.Rybak & Kochman-Kędziora, sp. nov. **A–O**LM images of valve views **P–AA**SEM images **P–T** images of entire valve external views **U–Z** view on valve central area and valve apices **AA** internal view of valve. Scale bars: 10 µm (**A–O**), 5 µm (**P, Q**), 4 µm (**R**), 3 µm (**S–U, Y–AA**), 2 µm (**V, W**), 1 µm (**X**).

**Description. LM** (Fig. 4A–O). Valves lanceolate to elliptical-lanceolate with convex valve margins. Valve dimensions (n = 25): length 7.5–14.0 µm, width 3.5–4.5 µm. Striae in light microscopy invisible. Raphe branches straight. Raphe located in elevated sternum, asymmetrically concave at the centre. Proximal raphe endings clearly visible, drop-like in shape and widely spaced. Distal raphe endings barely visible, curved in the same direction.

**Description. SEM** (Fig. 4P–AA). Longitudinal depressions present next to the sternum (Fig. 4P–U). Striae strongly radiating (36–42 in 10 µm), composed of one row of square to rounded areolae, externally not occluded, except central area (Fig. 4P–X). Central area large, lanceolate in shape, composed of fully externally silicified areolae composing striae (Fig. 4P–T, X). Raphe straight, filiform, located in raised and asymmetrically-constricted sternum (Fig. 4P–V, Y, Z). Proximal raphe fissures slightly asymmetrical drop-shape (Fig. 4U, Y, Z). Distal raphe fissures hooked and deflected to the same direction (Fig. 4V, X–Z). Transverse microcostae most prominent at the area near to valve margin (area of striae not covered by silica) (Fig. 4U–Y). A row of rounded to slightly elongated pores (3–5) present on the valve mantle, at the valve apices (Fig. 4U, Y, Z). Internally areolae occluded by hymenes. Raphe branches straight, forming small and elongated helictoglossae on distal endings, proximal endings drop-shaped. The central area is broad, corresponds in shape to external depression (Fig. 4AA).

### Species composition and diversity of moss-inhabiting diatom communities

**Figure 5.**
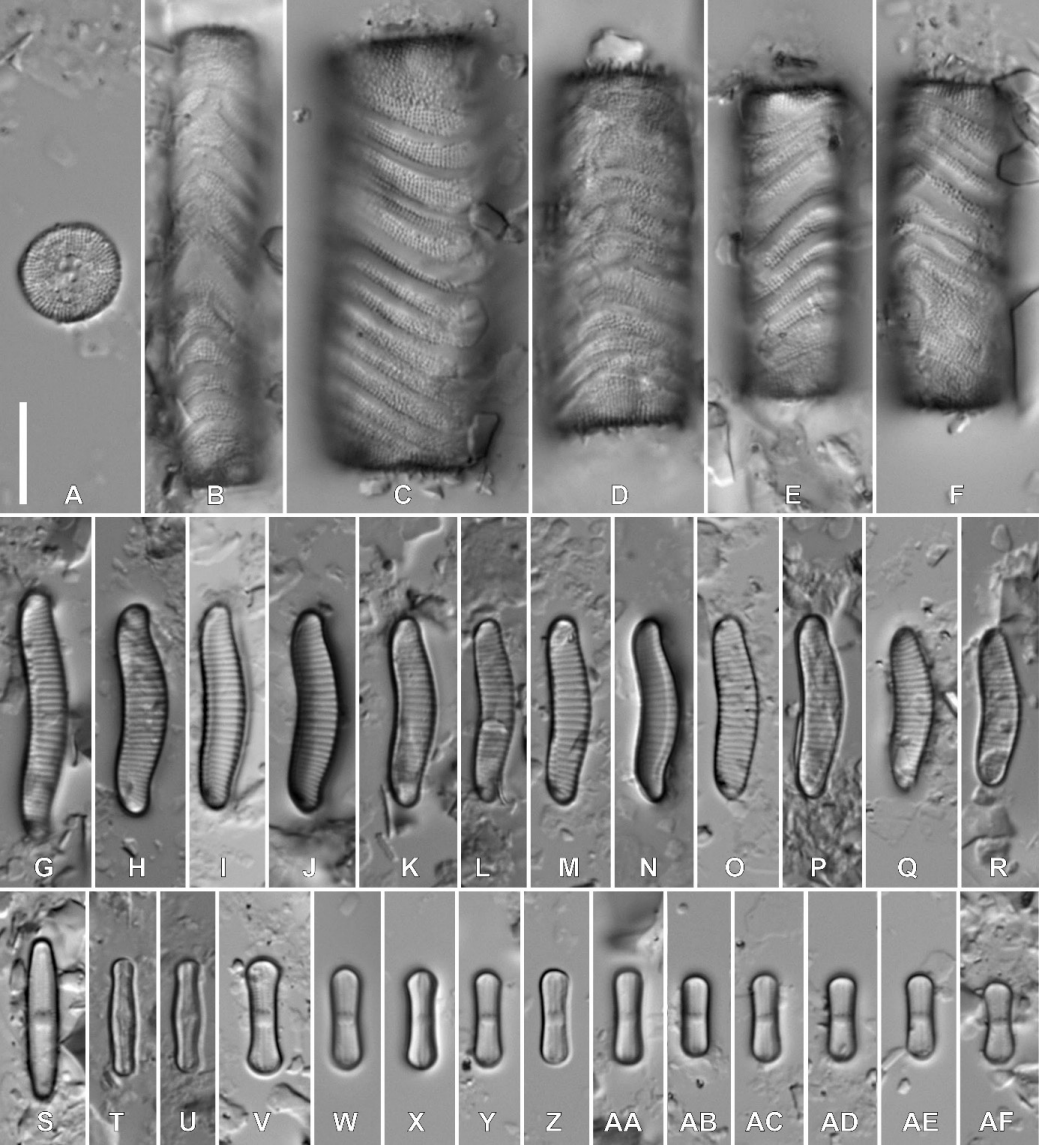
LM images of associated diatom flora **A–F***Orthoseiracircularis* (Ehrenberg) R.M. Crawford **G–R**Eunotiacf.pseudominor Pavlov & Levkov **S***Humidophila* sp. 1 **T, U***Humidophila* sp. 2 **V–A, F***Humidophilacontenta* (Grunow) Lowe et al. Scale bar: 10 µm.

**Figure 6.**
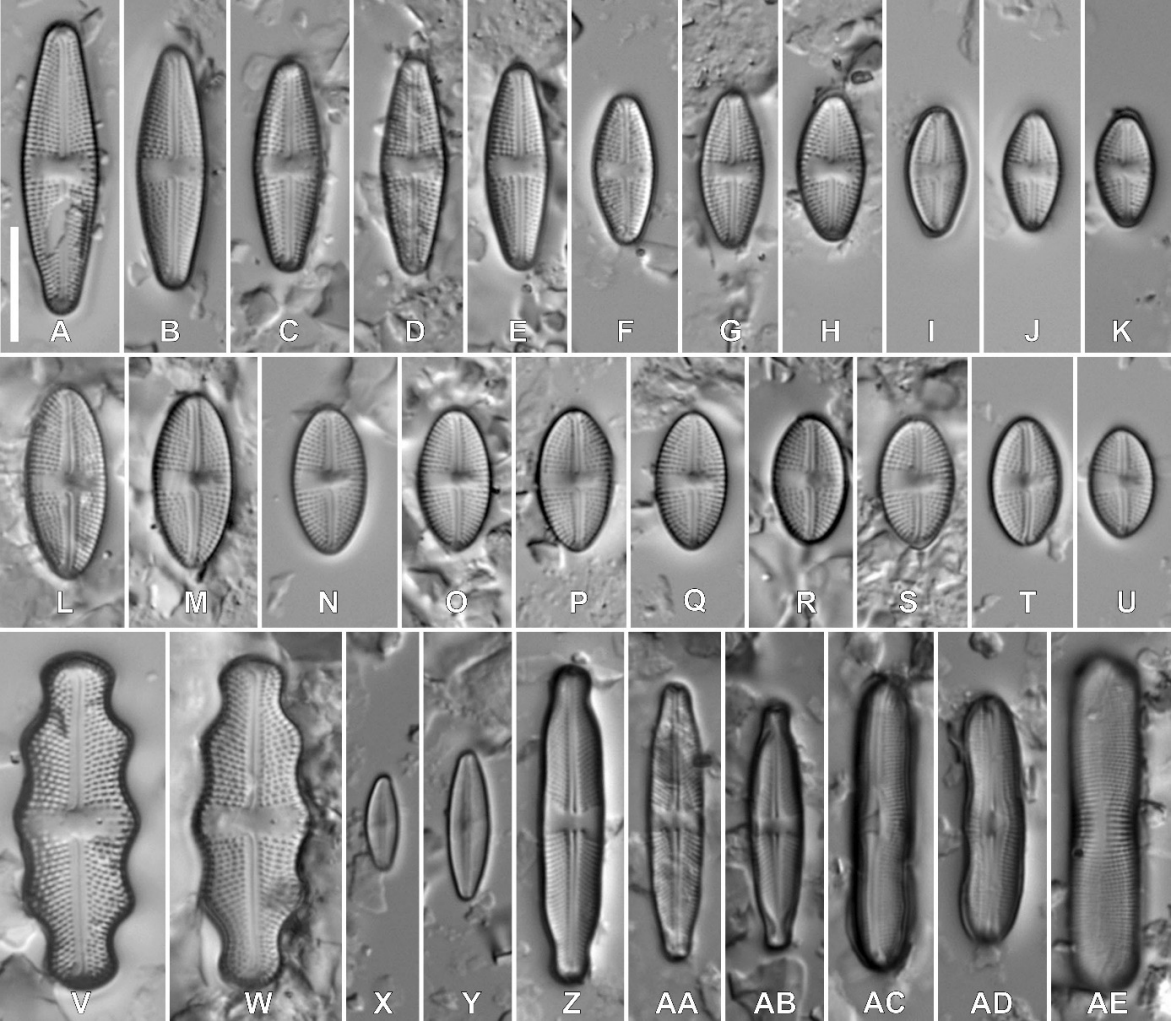
LM images of associated diatom flora **A–K***Luticolaintermedia* (Hustedt) Levkov, Metzeltin & Pavlov **L–U***Luticolapermuticoides* Metzeltin & Lange-Bertalot **V, W***Luticoladistinguenda* (Hustedt) Levkov, Metzeltin & Pavlov **X, Y***Nupelalesothensis* (Schoeman) Lange-Bertalot **Z–AB**Stauroneiscf.pygmaeaf.undulata Hustedt **AC–AE***Muelleria* sp. Scale bar: 10 µm.

**Figure 7.**
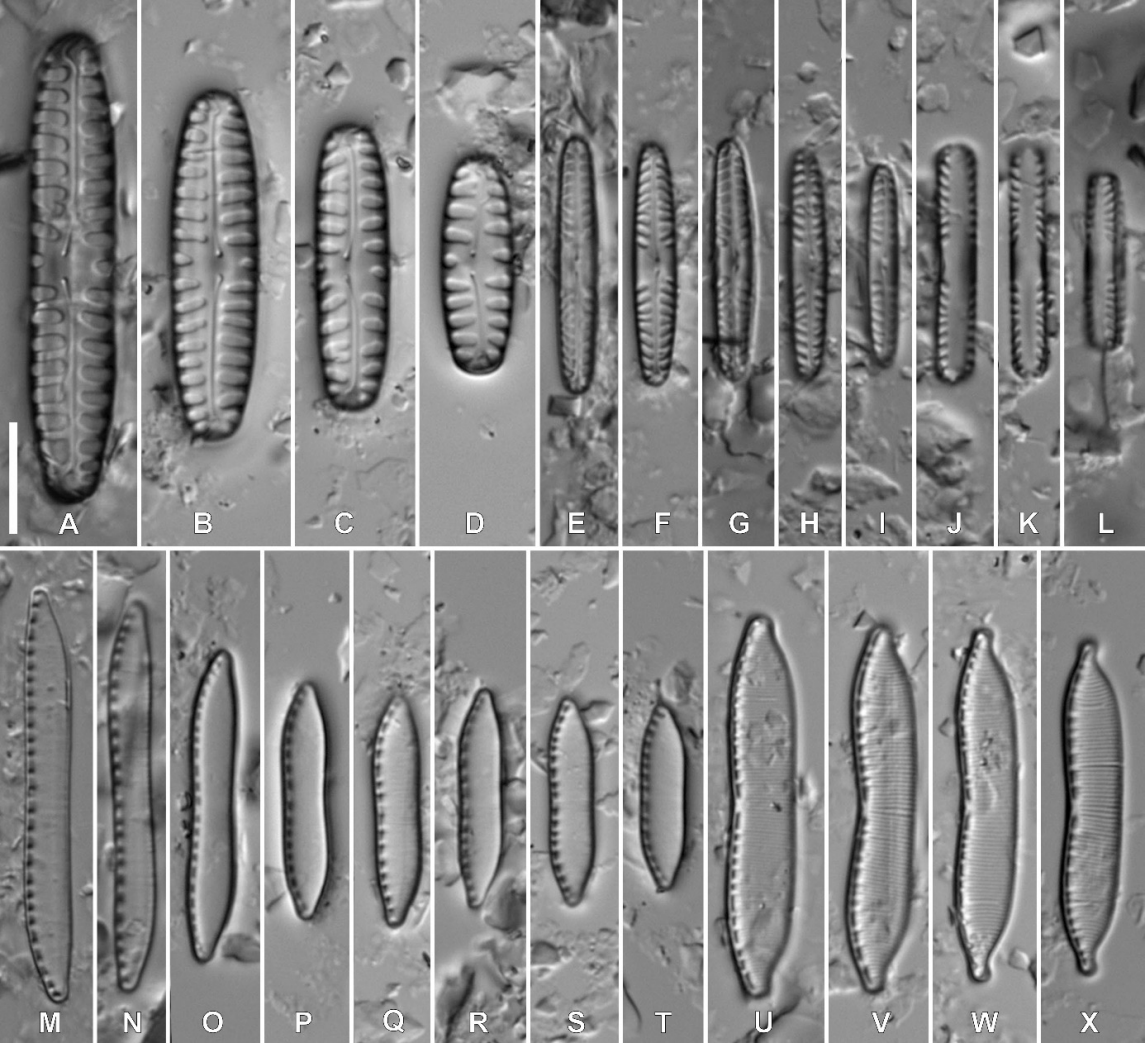
LM images of associated diatom flora **A–D***Pinnulariaborealis* Ehrenberg *sensu lato***E–L***Pinnularia* sp. **M–T***Nitzschiabrevissima* Grunow **U–X***Hantzschiaamphioxys* (Ehrenberg) Grunow. Scale bar: 10 µm.

**Figure 8.**
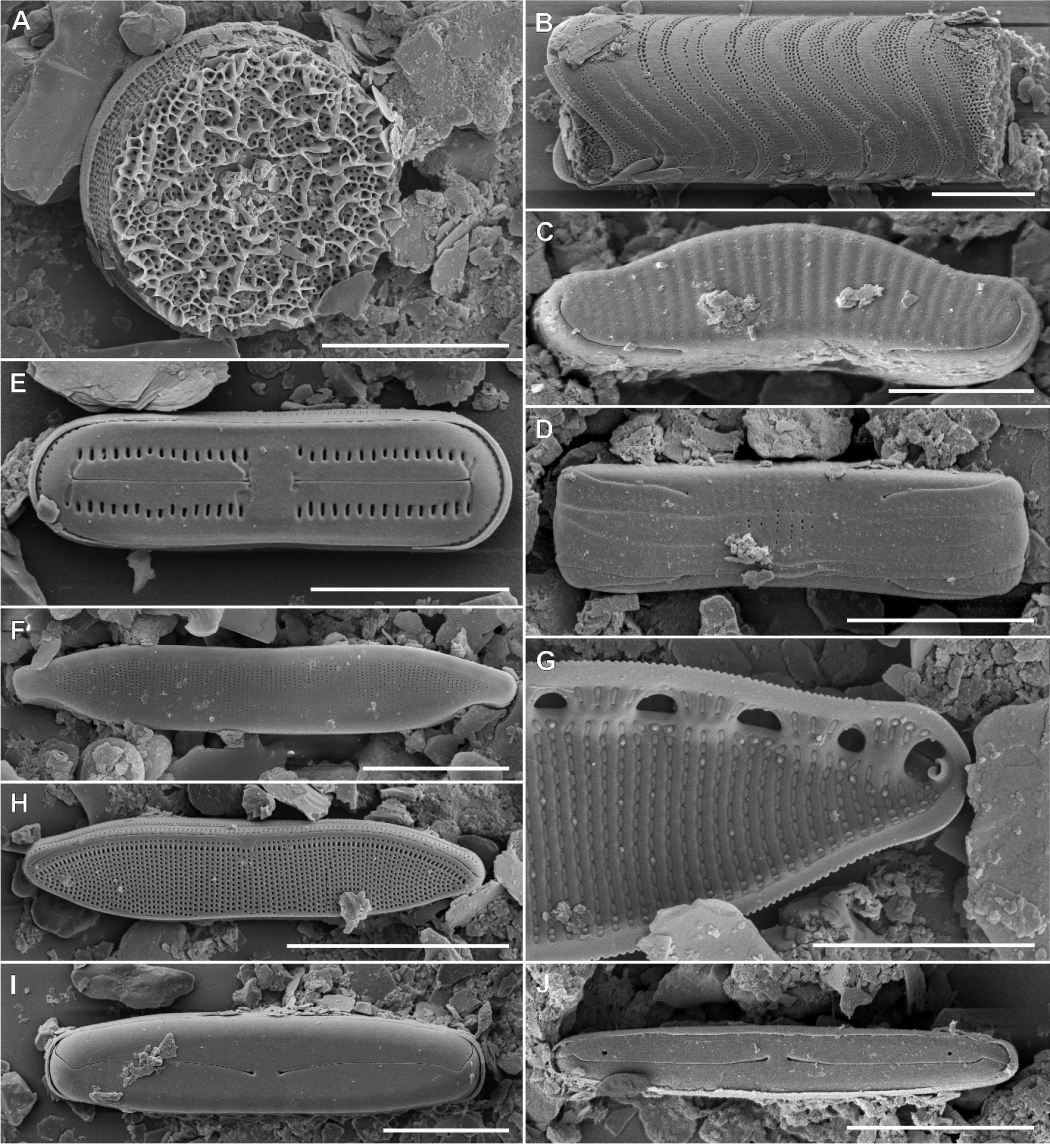
SEM images of associated diatom flora **A, B***Orthoseiracircularis* (Ehrenberg) R.M. Crawford **C, D**Eunotiacf.pseudominor Pavlov & Levkov **E***Humidophilacontenta* (Grunow) Lowe et al. **F***Hantzschiaamphioxys* (Ehrenberg) Grunow **G, H***Nitzschiabrevissima* Grunow **I***Pinnulariaborealis* Ehrenberg *sensu lato***J***Pinnularia* sp. Scale bars: 10 µm (**A, B, D, F, H, I, J**), 5 µm (**C**), 4 µm (**E**), 3 µm (**G**).

**Figure 9.**
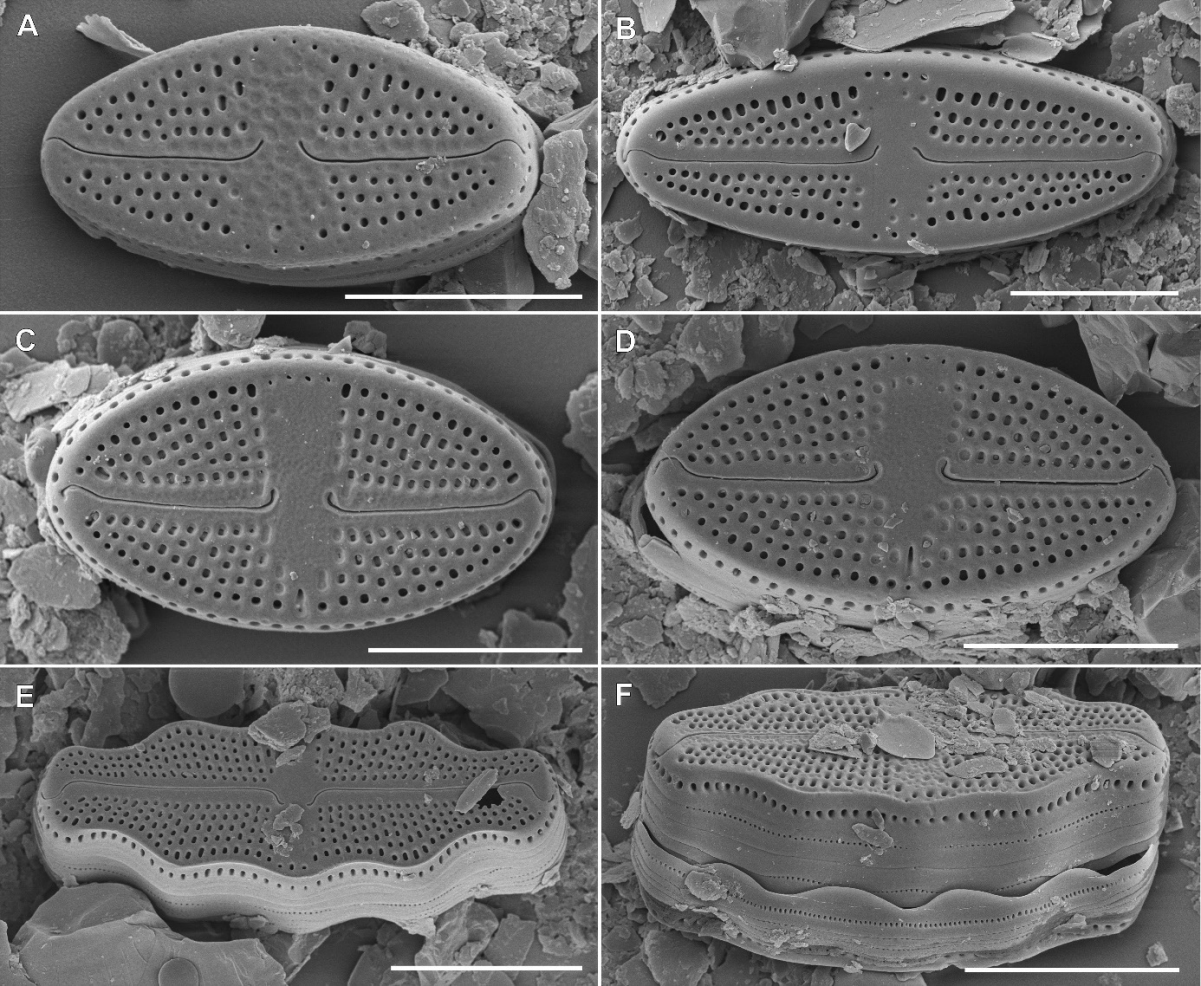
SEM images of associated diatom flora **A, B***Luticolaintermedia* (Hustedt) Levkov, Metzeltin & Pavlov **C, D***Luticolapermuticoides* Metzeltin & Lange-Bertalot **E, F***Luticoladistinguenda* (Hustedt) Levkov, Metzeltin & Pavlov. Scale bars: 15 µm (**A–D**), 10 µm (**E, F**).

A total of 20 diatom taxa were observed in all samples. Amongst them, four were described as a new species (Table 2, Figs 5–9).

**Table 2.** List of identified taxa together with dimensions and the percentage share in the assemblage. N – number of measured specimens.

**Taxa**	**N**	**Dimensions and stria density**			**Share in the assemblage** [%]		
		**Length [µm**]	**Width [µm**]	**Striae [in 10 µm**]	**Sample 2018/424**	**Sample 2018/425**	**Sample 2018/426**
***Eunotia*** aff. ***pseudominor*** Pavlov & Levkov	25	9.9–33.4	3.2–4.1	13–16	1.8	–	18.3
***Hantzschiaamphioxys*** (Ehrenberg) Grunow	25	29.6–43.1	4.6–5.7	26–30	2.4	< 0.1	8.4
***Humidophilacontenta*** (Grunow) Lowe, Kociolek, J.R. Johansen, Van de Vijver, Lange-Bertalot & Kopalová	15	6.2–11.1	2.1–2.8	ca. 40	57.7	62.1	1.5
***Humidophila*** sp. 1	5	11.6–17.7	2.5–3.1	38–39	< 0.1	< 0.1	–
***Humidophila*** sp. 2	5	9.6–11.7	2.4–2.7	–	–	–	< 0.1
***Luticolaasymmetrica* sp. nov.**	25	12.5–36.0	6.0–8.0	17–20	–	11.6	–
***Luticoladistinguenda*** (Hustedt) Levkov, Metzeltin & Pavlov	10	25.7–32.3	9.5–10.6	16–19	–	< 0.1	–
***Luticolaintermedia*** (Hustedt) Levkov, Metzeltin & Pavlov	25	9.4–25.6	4.6–6.6	20–24	9.4	ca. 1	–
***Luticolamicrocephala* sp. nov.**	25	14–24.0	4.5–6,6	19–22	6.6	–	12.6
***Luticolapermuticoides*** Metzeltin & Lange-Bertalot	25	6.3–20.4	5.1–7.4	19–23	–	12.5	–
***Luticolaterrestris* sp. nov**.	25	8.0–28.5	4.4–6.1	20–23	13.2	–	1.4
***Luticola*** cf. ***cristinae*** Levkov, Metzeltin & Pavlov	2	17–21.4	5.3–6.2	23–24	–	–	< 0.1
***Microcostatusmeridionalis* sp. nov.**	25	7.6–13.8	3.5–4.5	42–48	–	–	5.4
***Muelleria*** sp.	5	23.8–36.1	4.9–5.1	20–22	–	–	1.9
***Nitzschiabrevissima*** Grunow	15	15.6–41.1	3.7–4	40–43	7	< 0.1	21.8
***Nupelalesothensis*** (Schoeman) Lange-Bertalot	3	8.5–12.5	2.8–3.5	42	–	–	< 0.1
***Orthoseiracircularis*** (Ehrenberg) R.M. Crawford	10	Ø: 11.7–24.3		22–24	3.3	11.6	< 0.1
***Pinnulariaborealis*** Ehrenberg *sensu lato*	20	23–49.6	6.2–9.4	4.5–5	2.3	–	13.8
***Pinnularia*** sp.	10	15.7–24.8	2.8–3.3	5–6.5	–	–	3.8
***Stauroneis*** cf. ***pygmaea*** f. ***undulata*** Hustedt	15	19.7–34	3.6–5.1	21–25	–	–	7.6

## Discussion

The vast majority of research on diatoms in Africa was conducted in the second half of the twentieth century and many species descriptions are based on sketches and the classification of species is based on broad approaches to genera (Kociolek and Williams 2015). Many of them still wait for their verification using modern-day taxonomy focusing on detailed microscopic analysis.

Originally, many species of the genus *Luticola* were described as Navicula of section Punctatea. The genus verification made by Levkov et al. (2013) transferred most of them to *Luticola*, However, some taxa are still waiting for verification and potential transfer. Probably, based on sketches, the following species potentially are belonging to genus *Luticola*: Naviculalagerheimiif.rotundataCholnoky (1954, p. 218, pl. 3, fig. 81), Naviculainseratavar.ellipticaCholnoky (1960, p. 65, pl. 5, figs. 204–205), *Naviculasubmutica*Fusey (1964, p. 27, pl. 3, fig. 38), N.submuticavar.capitataFusey (1964, p. 27, pl. 3, fig. 41), N.submuticavar.ellipticaFusey (1964, p. 27, pl. 3, fig. 40), N.submuticavar.rectangularisFusey (1964, p. 27, pl. 3, fig. 39), *Naviculaguluensis*Giffen (1966, p. 238, fig. 70). However, their transfer require detailed study of type materials.

The three new *Luticola* species have comparable valve outline with protracted apices and striae density. They also share the presence of several shallow depressions in the central area visible in SEM. Despite their unique set of features, the taxa show some similarities to other species of this genus, especially under the light microscope. *Luticolamicrocephala* sp. nov. shows the highest degree of similarity to two aerophytic taxa described from India: *Luticolajogensis* (H.P. Gandhi) Kale, Levkov & Karthick (Kale et al. 2017, p. 30, figs 2–26) and *L.gandhii* Kale, Levkov & Karthick (Kale et al. 2017, p. 33, figs 27–51). However, *Luticolamicrocephala* sp. nov. can be easily separated from both Indian taxa. *Luticolajogensis* has much larger valves (28.5–36.0 µm length and 7.0–9.0 µm width versus 14.0–24.0 µm length and 4.5–6.6 µm width in *L.microcephala* sp. nov.). Additionally, *L.jogensis* has 5–6 areolae per striae, whereas striae in *L.microcephala* sp. nov. are composed of 3 to 4 areolae. The second of the Indian species *L.gandhii* differs from *L.microcephala* sp. nov. in having rostrate, not capitate apices. The most pronounced difference amongst all three species is the number of rows of areolae on the valve mantle. *Luticolamicrocephala* sp. nov. has only one row of elongated areolae, becoming more rounded in the central part of the valve and near the apices. On the contrary, *L.jogensis* has 2 or 3 rows (Kale et al. 2017, p. 34, fig. 22) and *L.gandhii* has always three rows of areolae (Kale et al. 2017, p. 37, fig. 48).

Amongst other species described from South Africa, Naviculasubmuticavar.capitata also shows some degree of similarity to *L.microcephala* sp. nov., but there is a lack of detailed microscopic pictures of this species. However, based on the description and the line drawing of this taxon (Fusey 1964, p. 27, pl. 3, fig. 41), Naviculasubmuticavar.capitata differs from *L.microcephala* sp. nov. in having more lanceolate valve outline, wider central area and lower striae density (16 in 10 µm versus 19–22 in *L.microcephala* sp. nov.).

*Luticolaasymmetrica* sp. nov. shows morphological similarity to five *Luticola* species reported from the African continent: *L.imbricatiformis* Levkov, Metzeltin & Pavlov (2013, p. 134, pl. 28, figs 1–11), *L.falknerorum* Metzeltin & Lange-Bertalot (Metzeltin and Lange-Bertalot 2007, p. 156, figs 1–9), *L.fuhrmannii* Metzeltin & Levkov in Levkov et al. (2013, p. 116, pl. 23, figs 1–20), *L.gesierichiae* Levkov, Metzeltin & Pavlov in Levkov et al. (2013, p.119, pl. 48, figs 17–28 and pl. 49, figs 1–5) and *L.frickei* Levkov, Metzeltin & Pavlov in Levkov et al. (2013, p. 114, pl. 48, figs 29–42, pl. 49, figs 6–8 and pl. 52, fig. 1). However, *Luticolaasymmetrica* sp. nov. can be distinguished, based on its asymmetrical, weakly dorsiventral valve margin. Additionally, *Luticolaimbricatiformis* has slightly elongated stigma positioned close to the centre of the valve (Levkov et al. 2013, p. 337, fig. 11), whereas *L.asymmetrica* sp. nov. has a round isolated pore located in the central area, halfway between the valve margin and central raphe. *Luticolafalknerorum* has a higher striae density (21–24 in 10 μm in comparison to 17–20 in 10 µm in *Luticolaasymmetrica* sp. nov.) and more areolae per striae (5–6 versus 3–5 in *Luticolaasymmetrica* sp. nov.). Both species can also be separated, based on external raphe structure. Raphe branches of *L.falknerorum* are bordered by silica ridges and have short proximal raphe endings (Levkov et al. 2013, p. 341 figs 2, 3), whereas *L.asymmetrica* sp. nov. does not have silica ridges and has asymmetrical, long proximal raphe endings, almost reaching the first row of areolae. Similar silica ridges are also present in *L.gesierichiae* (Levkov et al. 2013, p. 379, figs 2, 3). The characteristic asymmetrical valve outline with broadly-rounded apices is the main feature distinguishing *L.asymmetrica* sp. nov. from the mentioned *L.gesierichiae*, as well as from *L.frickei*. Both species have a regular linear-lanceolate valve shape with narrowly-rounded apices (Levkov et al. 2013). The last of the similar species – *Luticolafuhrmannii* is widely distributed in tropical areas of South America and Africa. Nonetheless, it can be separated, based on its elongated stigma positioned almost on the valve mantle (Levkov et. al. 2013, p. 385, fig. 4), whereas stigma of *Luticolaasymmetrica* sp. nov. are round and located on the central area in the middle between the valve margin and proximal raphe endings.

The third of described *Luticola* species – *Luticolaterrestris* sp. nov shows a high degree of similarity with several species from two informal morphological groups proposed by Levkov et al. (2013). *Luticolaterrestris* sp. nov resembles *L.tenuis* Levkov, Metzeltin & Pavlov (2013, p. 236, pl. 35, figs 1–18) and *L.micra* Levkov, Metzeltin & Pavlov (2013, p. 156, pl. 35, figs 19–37) from the group A and *L.incana* Levkov, Metzeltin & Pavlov from the group B. *Luticolatenuis* and *Luticolaterrestris* sp. nov. are observed from terrestrial habitats. Both species overlap dimensions; however, *Luticolaterrestris* sp. nov. has higher striae density (20–23 in 10 µm versus 18–20 in *L.tenuis*). Moreover, *L.tenuis* has more linear valve outline and slightly deflected proximal raphe endings (Levkov et al. 2013, p. 35, figs 17, 18) in contrast to more curved proximal raphe fissures in *L.terrestris* sp. nov. *Luticolamicra* differs from *Luticolaterrestris* sp. nov. in range of valve dimensions (8–18 µm length and 4–5 µm width versus 8.1–28.3 µm length and 4.4–6.1 µm width) and the lack of ghost areolae in the central area. Additionally, *L.micra* has elongated stigma (Levkov et al. 2013, p. 35, fig. 19), whereas stigma in *L.terrestris* sp. nov. are regularly rounded. In addition, *Luticolaincana* shows similarity with *Luticolaterrestris* sp. nov., especially in the valve outline; however, it has a smaller range of valve dimensions (12.5–20.5 µm length and 5–6 width vs. 8.1–28.3 µm length and 44.0–6.1 µm width). Moreover, *L.incana* does not show a ghost areolae in the central area and has short, slightly deflected distal raphe endings (Levkov et al. 2013, p. 135) in contrast to *L.terrestris* sp. nov., where distal raphe endings are distinctly hooked and expanded on to the valve mantle. Finally, all three compared species are described from other continents: both *L.micra* and *L.tenuis* from Europe and *L.incana* from South America. Amongst species described from Africa, two *Navicula* taxa (which probably should be placed in the genus *Luticola*) show some degree of similarity to *L.terrestris* sp. nov. *Naviculaguluensis* can be easily distinguished, based on striae density (15 in 10 µm versus 20–23 in 10 µm) and shape of raphe endings. *Naviculaguluensis* has shortly deflected distal raphe endings (Giffen 1963, fig. 60; Giffen 1966, pl 3, fig. 59), whereas distal raphe endings of *L.terrestris* sp. nov are elongated and reach the valve mantle. The second of the African species Naviculasubmuticavar.rectangularis shares with *L.terrestris* sp. nov. the valve outline, but has less dense striae with only 15–20 in 10 µm (Fusey 1964, p. 27, pl. 3, fig. 39).

Based on light microscopy observations, the *Microcostatusmeridionalis* sp. nov. is the most similar to *Microcostatusegregius* (Hustedt) Lange-Bertalot (Stanek-Tarkowska et al. 2016), *Microcostatuswerumii* Metzeltin, Lange-Bertalot & Soninkhishig (Metzeltin et al. 2009), *Microcostatusedaphicus* C.E. Wetzel, Noga, Ector & Stanek-Tarkowska (Stanek-Tarkowska et al. 2016), *Microcostatusaerophilus* Stanek-Tarkowska, Noga, C.E. Wetzel & Ector (Stanek-Tarkowska et al. 2016) and *Microcostatuskrasskei* (Hustedt) J.R. Johansen & Sray (Johansen and Sray 1998). All species share similar valve outline and invisible or very difficult to observed striae in LM. Only in two species – *M.meridionalis* sp. nov. and *M.werumii*, apart from a raphe and valve outline, no other morphological features are discernible in LM. In other species, some additional features (for example, depressions on both sides of the sternum, visible as arch-shaped shadows or striation pattern) can be observed in LM. *Microcostatusmeridionalis* sp. nov can be separated, based on its cuneate valve ends, whereas *M.werumii* has slightly rostrate valve ends. Additionally, proximal raphe endings are different in both species – in *M.werumii*, they are are poorly separated and located close to each other (Metzeltin et al. 2009, p. 225, figs 1–16). The drop-like proximal raphe endings of *M.meridionalis* are clearly visible, located on the asymmetrically constructed sternum. Under SEM, *M.meridionalis* sp. nov. poses a set of unique features that allows it to be easily distinguished from other representatives of the genus *Microcostatus*. *Microcostatusmeridionalis* sp. nov. has areolae occluded only in the central area, not on the entire valve face. Moreover, it has no pseudoconopeum/conopeum in contrast to *M.aerophilus* and *M.egregius* (Stanek-Tarkowska et al. 2016, p. 166, figs 13–15). Internally, in the aspect of striae pattern and the shape of the central area, the described species is similar to *Microcostatusschoemanii* Taylor, Levanets, S. Blanco & Ector (Taylor et al. 2010, p. 180, fig. 8) and *M.cholnokyi* Taylor, Levanets, S. Blanco & Ector (Taylor et al. 2010, p. 182, fig. 25), but, based on the different shape of raphe endings and valve outline, are easy to distinguish. Regarding valve dimensions and striae density, *Microcostatusmeridionalis* sp. nov. resembles *M.krasskei.* Both taxa overlap their dimensions (5–14 µm length, 3–4 µm width and 35–45 striae in 10 µm in *M.krasskei* versus 7.5–14 µm length, 3.5–4.5 µm width and 36–42 striae in 10 µm in *M.meridionalis*), but can be separated, based on the striae pattern and present of central area in. *M.meridionalis* (Johansen and Sray 1998, p. 99, figs 17, 19). The others of the aforementioned most similar species, although they have a similar shape, differ in striae density. *Microcostatusegregius* has 33–36 striae in 10 µm, whereas *Microcostatusaerophilus* up to 40–50 in 10 µm (Metzeltin et al. 2009; Stanek-Tarkowska et al. 2016), unlike to *Microcostatusmeridionalis* which has 36–42 striae in 10 µm. *Microcostatusaerophilus* (Stanek-Tarkowska et al. 2016) fully matches in the aspect of valve dimensions, but usually has higher striae density (40–50 in 10 µm).

The studied assemblages consisted of diatoms with various geographic distributions. Cosmopolitan species, such as: *Humidophilacontenta*, *Hantzschiaamphioxys*, *Nitzschiabrevissima*, *Pinnulariaborealis* (Krammer and Lange-Bertalot 1986; Krammer 2000; Lange-Bertalot et al. 2017) occurred together with species with a wide distribution in the tropics – *Luticoladistinguenda* and *L.intermedia* (Levkov et al. 2013; Glushchenko et al. 2017; Straube et al. 2017; Da Silva-Lehmkuhl et al. 2019). On the other hand, several of the noted species have not been reported from Africa so far or their occurrence in Africa has not been confirmed. *Luticolapermuticoides* was originally described by Metzeltin and Lange-Bertalot (2007) from South America. So far, the occurrence of this species in Africa has not been confirmed. However, in the original description, the authors indicated the presence of a similar taxon (Metzeltin and Lange-Bertalot 2002, plate 27, fig. 17) in Madagascar, which probably is, indeed, *Luticolapermuticoides*. Another species that has not been recorded in Africa so far is *Orthoseiracircularis*, a taxon originally described from South America, but observed also in materials from Java (Houk et al. 2017). Together with other species identified in our study, *O.circularis* is probably a typical inhabitant of the terrestrial environment. Based on the presented results and literature data, both mentioned taxa can be considered as having pantropical distribution in terrestrial habitats.

In the studied material, some species were scarce and their exact identification was not entirely possible. In the literature data, there is a lack of information about valve dimensions of Stauroneispygmaeaf.undulata – the species originally described from Asia (Hustedt 1942). For this reason, despite the high level of morphological similarity in the valve outline and striae pattern, the observed species were noted as Stauroneiscf.pygmaeaf.undulata. Two populations of *Eunotia*, observed in samples 2018/424 and 2018/426, show a highly similar pattern to *Eunotiapseudominor* Pavlov & Levkov. Both of our populations have similar dimensions: 12–35.6 µm length, 3.6–5.3 µm width and 11–15 striae in 10 µm in *E.pseudominor* (Pavlov and Levkov 2013) and 9.9–33.4 µm length, 3.2–4.1 µm width and 13–16 striae in 10 µm in our populations of *Eunotia* aff. *Pseudominor*; however, smaller valves were observed in our material. Moreover, the taxa observed in this study have less straight valve outline and more bent apices. Both taxa are also associated with moss vegetation. All of the observed differences in morphology can result from both the possible presence of two distinct species or only morphological differences within a single widely-distributed species. Therefore, we decided to identify it as similar to *E.pseudominor* (Eunotiaaff.pseudominor).

Many diatom taxa develop various adaptations to changes in humidity of aerial habitats, such as: production of thickened valves, reduction of areolae number and occlusion of areolae with silica (Lowe 2011; Lowe et al. 2007; Round et al. 1990; Dodd and Stoermer 1962; Main 2003). Newly-described species represent genera commonly noted in various terrestrial habitats (Johansen and Sray 1998; Levkov et al. 2013; Lange-Bertalot et al. 2017). Amongst them, the highest level of adaptation is shown in *M.meridionalis*, which has fully covered areolae in the central part of the valve. Accompanying *Pinnularia* and *Hantzschia* taxa also show the presence of external areolae occlusions. This type of structure is also present on the surface of Eunotiacf.pseudominor, whereas the genus *Eunotia* does not create hymen or other occlusions (Round et. al 1990). The development of diatoms as epiphytes on mosses, which are able to accumulate water to provide a favourable environment for many organisms, can by also considered as form of adaptation to local environmental conditions (Round 1957; Lindo and Gonzales 2010; Glime 2017).

The investigated moss samples were characterised by a small diversity of diatom species (from 9 to 15 species per sample). This low species richness is quite typical for terrestrial environments, such as soils, rock crevices or clumps of terrestrial bryophytes. Especially in the Southern Hemisphere, terrestrial diatom assemblages are still poorly known, both in taxonomic and ecological aspects. The present study showed that these environments are often “hot spots” for the occurrence of potentially new and rare taxa.

## Supplementary Material

XML Treatment for Luticola microcephala

XML Treatment for Luticola asymmetrica

XML Treatment for Luticola terrestris

XML Treatment for Microcostatus meridionalis
